# Increased RyR2 activity is exacerbated by calcium leak-induced mitochondrial ROS

**DOI:** 10.1007/s00395-020-0797-z

**Published:** 2020-05-22

**Authors:** Shanna Hamilton, Radmila Terentyeva, Benjamin Martin, Fruzsina Perger, Jiaoni Li, Andrei Stepanov, Ingrid M. Bonilla, Björn C. Knollmann, Przemyslaw B. Radwański, Sandor Györke, Andriy E. Belevych, Dmitry Terentyev

**Affiliations:** 1 Dorothy M. Davis Heart and Lung Research Institute, College of Medicine, The Ohio State University, Columbus, OH 43210 USA; 2 Department of Physiology and Cell Biology, College of Medicine, The Ohio State University, Columbus, OH 43210 USA; 3 Laboratory of Cell Pathology, Institute RAS, Saint Petersburg, Russia; 4 Division of Clinical Pharmacology, Vanderbilt University Medical School, Nashville, TN 37232 USA; 5 Division of Pharmacology, College of Pharmacy, The Ohio State University, Columbus, OH 43210 USA

**Keywords:** Ryanodine receptor, Ca^2+^ leak, Reactive oxygen species, Mitochondria, Ventricular arrhythmia, Catecholaminergic polymorphic ventricular tachycardia

## Abstract

Cardiac disease is associated with deleterious emission of mitochondrial reactive oxygen species (mito-ROS), as well as enhanced oxidation and activity of the sarcoplasmic reticulum (SR) Ca^2+^ release channel, the ryanodine receptor (RyR2). The transfer of Ca^2+^ from the SR via RyR2 to mitochondria is thought to play a key role in matching increased metabolic demand during stress. In this study, we investigated whether augmented RyR2 activity results in self-imposed exacerbation of SR Ca^2+^ leak, via altered SR-mitochondrial Ca^2+^ transfer and elevated mito-ROS emission. Fluorescent indicators and spatially restricted genetic ROS probes revealed that both pharmacologically and genetically enhanced RyR2 activity, in ventricular myocytes from rats and catecholaminergic polymorphic ventricular tachycardia (CPVT) mice, respectively, resulted in increased ROS emission under β-adrenergic stimulation. Expression of mitochondrial Ca^2+^ probe mtRCamp1h revealed diminished net mitochondrial [Ca^2+^] with enhanced SR Ca^2+^ leak, accompanied by depolarization of the mitochondrial matrix. While this may serve as a protective mechanism to prevent mitochondrial Ca^2+^ overload, protection is not complete and enhanced mito-ROS emission resulted in oxidation of RyR2, further amplifying proarrhythmic SR Ca^2+^ release. Importantly, the effects of augmented RyR2 activity could be attenuated by mitochondrial ROS scavenging, and experiments with dominant-negative paralogs of the mitochondrial Ca^2+^ uniporter (MCU) supported the hypothesis that SR-mitochondria Ca^2+^ transfer is essential for the increase in mito-ROS. We conclude that in a process whereby leak begets leak, augmented RyR2 activity modulates mitochondrial Ca^2+^ handling, promoting mito-ROS emission and driving further channel activity in a proarrhythmic feedback cycle in the diseased heart.

## Introduction

Contraction of the heart is a high-energy demanding process. Most of the energy required for this process in the form of ATP is generated by mitochondria, which occupy ~ 35% of myocyte volume [56]. In ventricular myocytes (VMs), release of Ca^2+^ from the sarcoplasmic reticulum (SR) by ryanodine receptors (RyR2s) is critical in initiating muscle contraction and a major determinant of its strength [24]. During SR Ca^2+^ release, local transfer of Ca^2+^ to the mitochondrial matrix activates enzymes in the tricarboxylic acid cycle and drives the electron transport chain (ETC) to accelerate ATP production [52], thereby providing a link between Ca^2+^-dependent contraction and mitochondria metabolic output. It is well established that in cardiac disease such as heart failure (HF), abnormal mitochondrial function is often accompanied by increased emission of reactive oxygen species (ROS) [9]. Excessive mitochondria-derived ROS (mito-ROS) evokes profound changes in intracellular Ca^2+^ homeostasis [47]. Importantly, a mito-ROS-mediated increase in activity of RyR2 has been linked to the increased propensity for aberrant Ca^2+^ leak from the sarcoplasmic reticulum (SR), leading to diminished systolic Ca^2+^ transients and increased incidence of pro-arrhythmic diastolic Ca^2+^ waves [18]. However, the role of increased RyR2-mediated Ca^2+^ release on SR-mitochondria Ca^2+^ transfer and mito-ROS emission remains incompletely understood.

Mitochondrial ATP production and SR Ca^2+^ release are closely coupled processes during the ‘fight-or-flight’ response, whereby β-adrenergic receptors are stimulated due to an increased requirement of the heart to meet metabolic demand. A positive inotropic effect of the pathway includes augmentation of RyR2 activity, increasing the availability of cytosolic Ca^2+^ to activate the contractile machinery [7]. However, as β-adrenergic drive can exacerbate Ca^2+^ leak from RyR2 channels that are already hyperactive in cardiac disease, the detrimental effects of excessive diastolic Ca^2+^ leak are most obvious during this response. Leak of Ca^2+^ from the SR can precipitate to cause triggered activity and many life-threatening cardiac arrhythmias [8, 42, 68]. This is particularly evident in catecholaminergic polymorphic ventricular tachycardia (CPVT), a genetic disorder characterized by mutations in the RyR2 macromolecular complex, whereby ventricular tachycardia and fibrillation (VT/VF) often only present in patients after exercise or stress [30, 57].

It has also been suggested that during β-adrenergic stimulation, increased energy demand directly drives mito-ROS emission, thus enhancing aberrant SR Ca^2+^ leak via oxidized RyR2 [12–14]. When oxidative phosphorylation and electron flux via the ETC are accelerated to produce more ATP, a higher rate of electron slippage can occur, with the formation of mito-ROS as a consequence [9]. Enhancement of mitochondrial Ca^2+^ (mito-Ca^2+^) levels has previously been associated with increased mito-ROS production [27, 51, 59, 60], while in failing guinea pig hearts, others have observed decreased mito-Ca^2+^ levels in parallel with elevated mito-ROS emission [38, 46, 48]. We recently demonstrated that pharmacological enhancement of mito-Ca^2+^ accumulation leads to increased oxidation of RyR2 [32]. In the present study, we test the hypothesis that augmented RyR2-mediated SR Ca^2+^ leak feeds back on the oxidation status of RyR2 via mito-ROS, thus further exacerbating leak.

Using fluorescent indicators and genetically encoded spatially restricted ROS biosensors, we demonstrate that under conditions of both pharmacologically and genetically increased RyR2 activity, β-adrenergic stimulation by isoproterenol (ISO) increases ROS emission in VMs, both at the outer mitochondrial membrane and in close vicinity to RyR2 within the SR. Live cell imaging of mito-Ca^2+^ dynamics showed that acute enhanced RyR2-mediated Ca^2+^ leak resulted in diminished ability of mitochondria to accumulate and retain Ca^2+^. This led to oxidation of RyR2, further exacerbating SR Ca^2+^ leak. Importantly, augmented leak and the resulting decrease in Ca^2+^ transient amplitude, as well as the increase in incidence of proarrhythmic spontaneous Ca^2+^ waves (SCWs), were attenuated by scavenging of mito-ROS. Transfer of Ca^2+^ from the SR to mitochondria is essential for the enhancement of mito-ROS emission, as demonstrated by experiments with dominant-negative paralogs of the mitochondrial Ca^2+^ uniporter (MCU). Our results suggest that increased RyR2 activity disturbs mito-Ca^2+^ homeostasis and elevates mito-ROS emission, thus in a vicious feedback cycle exacerbates diastolic SR Ca^2+^ leak by increasing RyR2 oxidation, driving spontaneous Ca^2+^ release that is detrimental in cardiac disease.

## Methods

### Production of adenoviral constructs

The intra-mitochondrial Ca^2+^ biosensor mtRCamp1h was constructed by fusing cytochrome C oxidase subunit IV at the N-terminus of RCamp1h coding region [1], as described previously [32]. The outer-mitochondrial membrane H_2_0_2_ biosensor OMM-HyPer was constructed by fusing mAKAP1 followed by a linker to the N-terminus of the coding region of pC1-HyPer-3 [10], as described previously [32]. Oxidation in the SR was measured using the ERroGFP_iE biosensor [2], as described previously [32]. The pCMV G-CEPIA1er biosensor was a gift from Masamitsu Iino (Addgene plasmid # 58,215) [63].

Adenoviruses carrying biosensor constructs were generated utilizing the ViraPower Gateway expression system (Thermo Fisher Scientific, Waltham, MA, USA). Coding regions of described plasmids were cloned into the pENTR™ 1A entry vector and recombined into the pAd/CMV/V5-DEST™ destination vector by LR recombinase reaction. After sequence verification, destination vectors were digested with restriction endonuclease *PacI*, before transfection into HEK293A cells using Lipofectamine™ 2000 (Thermo Fisher Scientific) for viral production.

The MCU-DN adenovirus was kindly gifted by Dr. Jin O-Uchi, University of Minnesota Twin Cities.

### Study animals

#### Control rats

Male Sprague–Dawley rats (controls) were obtained from Charles River Laboratories. Rats were aged 8–12 weeks. A total of 48 rats were used for the study.

#### Wild-type and calsequestrin-null mice

Male and female wild-type (WT) and calsequestrin2-null mutant mice in the C57BL/6 genetic background [37] were utilized in this study, and are referred to as CPVT mice. Mice were aged 8–14 months. A total of 16 mice were used for the study.

### Myocyte isolation

#### Rat VM isolation

Ventricular myocytes were obtained by enzymatic digestion as previously described [32]. Rats were anesthetized with a lethal dose of sodium pentobarbital solution (120 mg/kg) before the heart was rapidly removed. Hearts were immersed in cold Ca^2+^-free Tyrode’s solution (in mmol/L: 140 NaCl, 5.4 KCl, 1.0 CaCl_2_, 1 MgCl_2_, 10 HEPES, 5.6 glucose, pH 7.3). Hearts were then mounted on a Langendorff apparatus and retrogradely perfused through the aorta with Tyrode’s solution containing collagenase II (Worthington Biochemical Corp.) at 37 °C for 16 min. Ventricles were minced and placed in a 37 °C water bath shaker in collagenase solution. Isolated VMs were then prepared for primary culture.

#### Mouse VM isolation

As previously described [31, 44], VMs from mice were also obtained by enzymatic digestion. Mice were anesthetized with isoflurane, hearts were rapidly excised, immersed in cold Ca^2+^-free Tyrode’s solution, and mounted on a Langendorff apparatus. Hearts were perfused with Tyrode’s solution containing liberase TH Research Grade enzyme (Roche) for 7 min. Ventricles were minced and single VMs were stabilized in perfusion solution containing BSA (20 mg/mL). Isolated VMs were plated onto laminin-coated glass coverslips in 24-well dishes in Tyrode’s solution containing 0.5 mmol/L Ca^2+^ and used immediately for imaging experiments.

### Primary culture of rat VMs

For experiments with cultured control rat VMs, myocytes were plated onto laminin-coated glass coverslips in 24-well dishes in serum-free medium 199 (Thermo Fisher Scientific), supplemented with 25 mmol/L NaHCO_3_, 10 mmol/L HEPES, 5 mmol/L creatine, 5 mmol/L taurine, 10 μ/mL penicillin, 10 μg/mL streptomycin and 10 μg/mL gentamycin (pH 7.3). Any unattached cells were removed by replacing the medium after 1 h. Myocytes were infected with adenoviruses and were cultured at 37 °C in 95% air and 5% CO_2_ for 36–48 h before analysis. Rat VMs maintain structural integrity including T-tubule organization and electrical properties for at least the first 48 h of culture [3].

### Confocal imaging

Laser scanning confocal imaging was performed using Leica SP8 dmi8 and Olympus Fluoview 1000 microscopes in x–y and linescan modes. Myocytes were paced via field stimulation using extracellular platinum electrodes.

#### Measurement of [Ca^2+^]_SR_ using G-CEPIA1er

To directly assess SR Ca^2+^ concentration ([Ca^2+^]_SR_) and RyR2-mediated SR Ca^2+^ leak, intact VMs were infected and cultured with G-CEPIA1er virus on glass coverslips. After 36–48 h, VMs were perfused with Tyrode’s solution containing 1 mmol/L Ca^2+^. Isoproterenol (ISO, 50 nmol/L) was added to the solution to stimulate β-adrenergic receptors. Low-dose caffeine (200 µmol/L) was added to induce RyR2 leak. Myocytes were preincubated with specific mitochondrial superoxide scavenger mitoTEMPO (20 μmol/L; Millipore Sigma) for 10 min prior to experimentation, and was included in the perfusion solution. Biosensor G-CEPIA1er was excited using 488 nm line of argon laser and fluorescence emission was collected at 500–550 nm, measured in *x*–*y* mode at 400 Hz sampling rate. Resting VMs were exposed to sarco/endoplasmic reticulum Ca^2+^-ATPase (SERCa2a) inhibitor thapsigargin (10 μmol/L) after 5 min in ISO or ISO and caffeine, and fluorescence signal from G-CEPIA1er was monitored using confocal microscopy. The time constant of decay of G-CEPIA1er was used as a measure of the leak by fitting fluorescence data to a monoexponential function [4]. The SR Ca^2+^ store was depleted by application of high-dose caffeine (10 mmol/L) in Ca^2+^-free Tyrode’s solution.

#### Standard pacing protocol for measurements using biosensors and indicators in rat VMs

This standard pacing protocol was followed during assays using OMM-HyPer, ERroGFP_iE, mtRCamp1h, Fluo-3 and TMRM. Baseline myocyte fluorescence was recorded for 5 min (0–5 min of recording) under continuous perfusion with Tyrode’s solution containing 1 mmol/L Ca^2+^. Myocytes were field stimulated for 5 min at 2 Hz (5–10 min of recording). At 12 min, ISO (50 nmol/L) or ISO plus low-dose caffeine (200 µmol/L) was added and continuously perfused (12–17 min of recording). Next, VMs were paced for 5 min during drug perfusion (17–22 min of recording). Following cessation of pacing, fluorescence was recorded for an additional 5 min before any further treatment, as described in each assay below.

#### Measurement of oxidative stress using ERroGFP_iE and OMM-HyPer

Oxidative stress in intact VMs within the SR and at the OMM was assessed using ERroGFP_iE and OMM-HyPer biosensors, respectively. Myocytes were infected with viruses on glass coverslips and cultured for 36–48 h, before perfusion with Tyrode’s solution (1 mmol/L Ca^2+^). Biosensors were excited using 488 nm line of argon laser and fluorescence emission was collected at 500–550 nm wavelengths, measured in the *x*–*y* mode at 400 Hz sampling rate. The pacing protocol was followed as described above. Minimum fluorescence was obtained by application of ROS scavenger dithiothreitol (DTT, 5 mmol/L), and maximum fluorescence (*F*_max_) was obtained by application of deoxythymidine diphosphate (DTDP, 200 µmol/L). Data are presented as a percentage of Δ*F*/Δ*F*_max_. where Δ*F* = *F*– *F*_min_ and Δ*F*_max_ = *F*_max_–*F*_min_.

#### Measurement of mito-[Ca^2+^] using mtRCamp1h

To assess mito-Ca^2+^ handling in intact VMs, cells were infected with mtRCamp1h virus on glass coverslips and cultured for 36–48 h. Myocytes were perfused with Tyrode’s solution (1 mmol/L Ca^2+^). Biosensor mtRCamp1h was excited using 543 nm line of HeNe laser and fluorescence emission was collected at 560–660 nm wavelengths, measured in the *x*–*y* mode and 400 Hz sampling rate. The pacing protocol was followed as described above. After this protocol, VMs were washed in Ca^2+^-free Tyrode’s solution, before permeabilization with saponin (0.001%). The solution was replaced with an internal recording solution containing cytochalasin D (10 μmol/L) and Ca^2+^ buffer EGTA (2 mmol/L) to obtain minimum mtRCamp1h fluorescence. Maximum fluorescence was achieved by application of Ca^2+^ (20 μmol/L). Using the equation [Ca^2+^]_m_ = Kd ×  (*F* – *F*_min_)/(*F*_max_ – *F*), where Kd of mtRCamp1h = 1.3 µmol/L, fluorescence was converted to [Ca^2+^]_m_ for each myocyte. Analysis parameters included peak mtRCamp1h [Ca^2+^]_m_ (μmol/L), the time to peak amplitude (s), the first derivative of [Ca^2+^]_m_ [62, 65] (nmol/L Ca^2+^ s^−1^) and the rate of decay (s^−1^).

#### Cytosolic Ca^2+^ imaging of rat VMs using Fluo-3 AM

To measure cytosolic Ca^2+^, cultured rat VMs were loaded with Fluo-3 AM (Invitrogen) at room temperature for 10 min in Ca^2+^-free Tyrode’s solution, followed by a 8 min wash in Tyrode’s solution containing 1 mmol/L Ca^2+^. Myocytes were perfused with Tyrode’s solution containing 1 mmol/L Ca^2+^ at room temperature during recordings. Fluo-3 AM was excited at 488 nm and fluorescence emission was collected at 500–550 nm wavelengths in line scan mode at 200 Hz sampling rate. The pacing protocol was followed as described above, with pacing for 5 min at 2 Hz, followed by the addition of either ISO (50 nmol/L) or ISO and low-dose caffeine (200 µmol/L), then further pacing for 5 min. For experiments with mitoTEMPO, VMs were preincubated with mitoTEMPO (20 μmol/L, Millipore Sigma) for 10 min prior to experimentation, and mitoTEMPO was included in the perfusion solution. Line scans were recorded during the last minute of pacing. Cytosolic Ca^2+^ transient amplitude is presented as Δ*F*/Δ*F*_0,_ where *F*_0_ is basal fluorescence and Δ*F* = *F*–*F*_0_.

#### Cytosolic Ca^2+^ imaging of mouse VMs using Fluo-3 AM

To measure intracellular Ca^2+^ transient, intact VMs were loaded with Fluo-3 AM (Invitrogen) at room temperature for 20 min in Tyrode’s solution containing 0.5 mmol/L Ca^2+^, followed by a 20 min wash in Tyrode’s solution containing 1 mmol/L Ca^2+^. Myocytes were then perfused with Tyrode’s solution containing 2 mmol/L Ca^2+^ and ISO (100 nmol/L) at room temperature during Ca^2+^ transient recordings. For experiments with mitoTEMPO, VMs were preincubated with mitoTEMPO (20 μmol/L, Millipore Sigma) for 20 min prior to experimentation, and mitoTEMPO was included in the perfusion solution. Fluo-3 AM was excited at 488 nm and fluorescence emission was collected at 500–550 nm wavelengths in line scan mode at 200 Hz sampling rate. To test for the propensity of triggered activity, VMs were stimulated for 10 s at 0.5 Hz and the latency between the last pacing stimulus and the subsequent SCW was calculated. To assess SR Ca^2+^ load, high-dose caffeine (10 mmol/L) was applied at the end of the experiments. The data are presented as Δ*F*/*F*_0_, where *F*_0_ is the basal fluorescence and Δ*F* = *F*–*F*_0_.

#### Measurement of mitochondrial superoxide in mouse VMs using MitoSOX

The emission of mitochondrial superoxide was measured in isolated VMs in Tyrode’s solution containing 2 mmol/L Ca^2+^ using MitoSOX Red mitochondrial superoxide indicator (Thermo Fisher Scientific; 20 μmol/L, 20 min loading). The indicator was excited with 514 nm line of an argon laser and emission was collected at 560–660 nm, measured in the *x*–*y* mode. Fluorescence of MitoSOX was normalized to the maximum fluorescence signal obtained by application of DTDP (200 µmol/L).

### Western blotting and assessment of RyR2 oxidation

#### List of antibodies used is present in Table 1

**Table 1:** Antibodies used in the study

Antibody/kit	Species of used sample	Source	Identifier
Anti-RyR2	Rat	Thermo Fisher Scientific	Cat#MA3-916
Anti-mouse IgG(H + L), HRP	Rat	Promega	Cat#W4021
Oxidized Protein Western blot kit	Rat	Abcam	Cat#ab178020
Anti-MCU	Rat and Mouse	Sigma-Aldrich	Cat#HPA016480
Anti-GAPDH	Rat and Mouse	Abcam	Cat#ab8245
Anti-Rabbit IgG(H + L),HRP	Rat	Promega	Cat#W4011
Anti-Mouse IgG(H + L) Alexa Fluor 633	Rat	Thermo Fisher Scientific	Cat#A-21052
Anti-RyR2	Mouse	Alomone	Cat#ARR-002
Anti-CCDC109B/MCUb (C-terminal)	Mouse	Abcam	Cat#ab170715

### Immunofluorescence for biosensor colocalization in rat VMs

Cultured control rat VMs plated on laminin-coated glass coverslips were infected with G-CEPIAer or ERroGFP_iE adenoviruses and were cultured at 37 °C in 95% air and 5% CO_2_ for 36–48 h. Myocytes were then prepared for immunofluorescence by fixing with 4% paraformaldehyde and permeabilized with 0.2% Triton X-100/PBS (pH 7.2) containing 1% BSA. Samples were then probed using RyR2 primary antibody. Secondary antibody used was goat anti‐mouse IgG (H + L) cross‐adsorbed secondary antibody Alexa Fluor 633.

### Staining with MitoTracker Red for biosensor colocalization in rat VMs

Cultured control rat VMs plated on laminin-coated glass coverslips were infected with OMM-HyPer adenovirus and were cultured at 37 °C in 95% air and 5% CO_2_ for 36–48 h. Live VMs were then stained with MitoTracker Deep Red FM (Invitrogen, 500 nM) for 15 min, then washed in Tyrode’s solution prior to imaging. The indicator was excited with 546 nm line of an argon laser and emission was collected at 550–660 nm, measured in the *x*–*y* mode.

### RyR2 immunoprecipitation and immunoblotting from rat VMs

Freshly isolated rat VMs were treated with isoproterenol (50 nmol/L),and caffeine (200 μmol/L) for 4 min prior to 2 Hz pacing for 1 min at room temperature. Cells were then immediately lysed in lysis buffer from Cell Signaling (Cat#9803S), supplemented with phosphatase (Calbiochem, Cat#524,625) and protease inhibitor cocktails (Sigma, Cat#P8340) as described previously (Terentyev et al. 2014).

RyR2 was immunoprecipitated from cell lysate using anti-RyR2 antibody (5 μL) in 0.5 mL RIPA buffer overnight at 4 °C. Samples were incubated with Protein A/G Plus-agarose beads (Santa Cruz cat # sc-2003) for 1 h at 4 °C and washed three times with RIPA buffer.

To determine the oxidation status of RyR2, the Oxidized Protein Western Blot Kit was used, whereby carbonyl groups of immunoprecipitated RyR2 were derivatized to 2,4 dinitrophenylhydrazone (DNP) by reaction with 2,4 dinitrophenylhydrazine. For control, we used the kit-provided Derivatization Control Solution. The DNP-RyR2 protein samples were separated on 4–20% Mini-PROTEAN TGX gels (Bio-Rad Laboratories, Cat#456–1094) and DNP-associated signal was assessed by the kit-provided anti-DNP rabbit primary antibody and anti-RyR2, followed by HRP-conjugated anti-rabbit goat secondary antibody and anti-mouse lgG(H + L),HRP secondary antibody.

Blots were developed with ECL (Bio-Rad Laboratories) and quantified using Image J (US National Institutes of Health) and Origin 8 software.

### RyR2 immunoprecipitation and immunoblotting from mouse VMs

Hearts were rapidly excised from isoflurane-anesthetized mice and immersed in cold Ca^2+^-free Tyrode’s solution before mounting on a Langendorff apparatus. Hearts were then perfused with Tyrode’s solution containing 2 mmol/L Ca^2+^ for 15 min. The atria were removed and ventricles were homogenized using Tissue Tearer Model 985,370 in 1 mL of buffer containing: Tris–HCL (50 mmol/L, pH 7.4), NaCl (150 mmol/L) NaF (5 mmol/L), Na_3_VO_4_ (1 mmol/L), 0.5% Triton-X 100, protease inhibitor and phosphatase inhibitor. The homogenate was centrifuged at 12000x*g* for 15 min at 4 °C. RyR2 was immunoprecipitated from the supernatant (500 mL) using anti-RyR2 antibody (3 µL) for 2 h at 4 °C. The samples were then incubated with Protein A/G Plus-agarose beads (Santa Cruz, Cat#sc-2003) for 1 h at 4 °C and washed three times with RIPA buffer.

To determine the oxidation status of RyR2, the Oxidized Protein Western Blot Kit was used, whereby the carbonyl groups of immunoprecipitated RyR2 were derivatized to DNP by reaction with 2,4dinitrophenylhydrazine. Kit-provided Derivatization Control Solution was used as control. The DNP-RyR2 protein samples were separated on 4–20% Mini-PROTEAN TGX gels and DNP-associated signal was assessed by the kit-provided anti-DNP rabbit primary antibody and anti-RyR2, followed by HRP-conjugated anti-rabbit goat secondary antibody and anti-mouse lgG (H + L), HRP secondary antibody.

Blots were developed with ECL (Bio-Rad Laboratories) and quantified using Image J (US National Institutes of Healthand Origin 8 software.

### Expression of MCU complex proteins

To determine MCU-DN expression, intact rat VMs were infected and cultured with MCU-DN virus on glass coverslips for 36–48 h. For the assessment of MCU/MCUb expression, freshly isolated mouse VMs from WT and CPVT hearts were obtained as described above.

Myocytes were lysed in lysis buffer from Cell Signaling (Cat#9803S), supplemented with phosphatase (Calbiochem, Cat#524,625) and protease inhibitor cocktails (Sigma, Cat#P8340) as described previously [36]. Samples (20–30 μg of proteins) were resolved on a 4–20% gel via SDS‐PAGE, transferred onto nitrocellulose membranes, and probed with antibodies specific for MCU complex proteins and subsequently probed with secondary antibody. Blots were developed with ECL (Bio‐Rad Laboratories) and quantified using ImageJ and Origin 8 software.

### Statistics

All statistical analysis was performed using Origin 8 (OriginLab). Data are presented as mean ± standard error (SEM). Uppercase *n*(*N*) = number of animals, and lowercase *n* = number of VMs. Statistical significance between groups was calculated using Student’s *t* test (paired and unpaired), Fisher’s exact test and one-way ANOVA with Bonferroni post hoc test, where appropriate. A *p* value of less than 0.05 was considered significant.

## Results

### Caffeine-mediated enhancement of SR Ca^2+^ leak is reduced by mito-ROS scavenging

When used at low concentrations, caffeine acts as an agonist of RyR2. We first sought to demonstrate that low-dose caffeine (200 µmol/L) could reproduce the leaky SR phenotype observed in cardiac disease, using cultured VMs isolated from healthy rat hearts.

**Fig.1:**
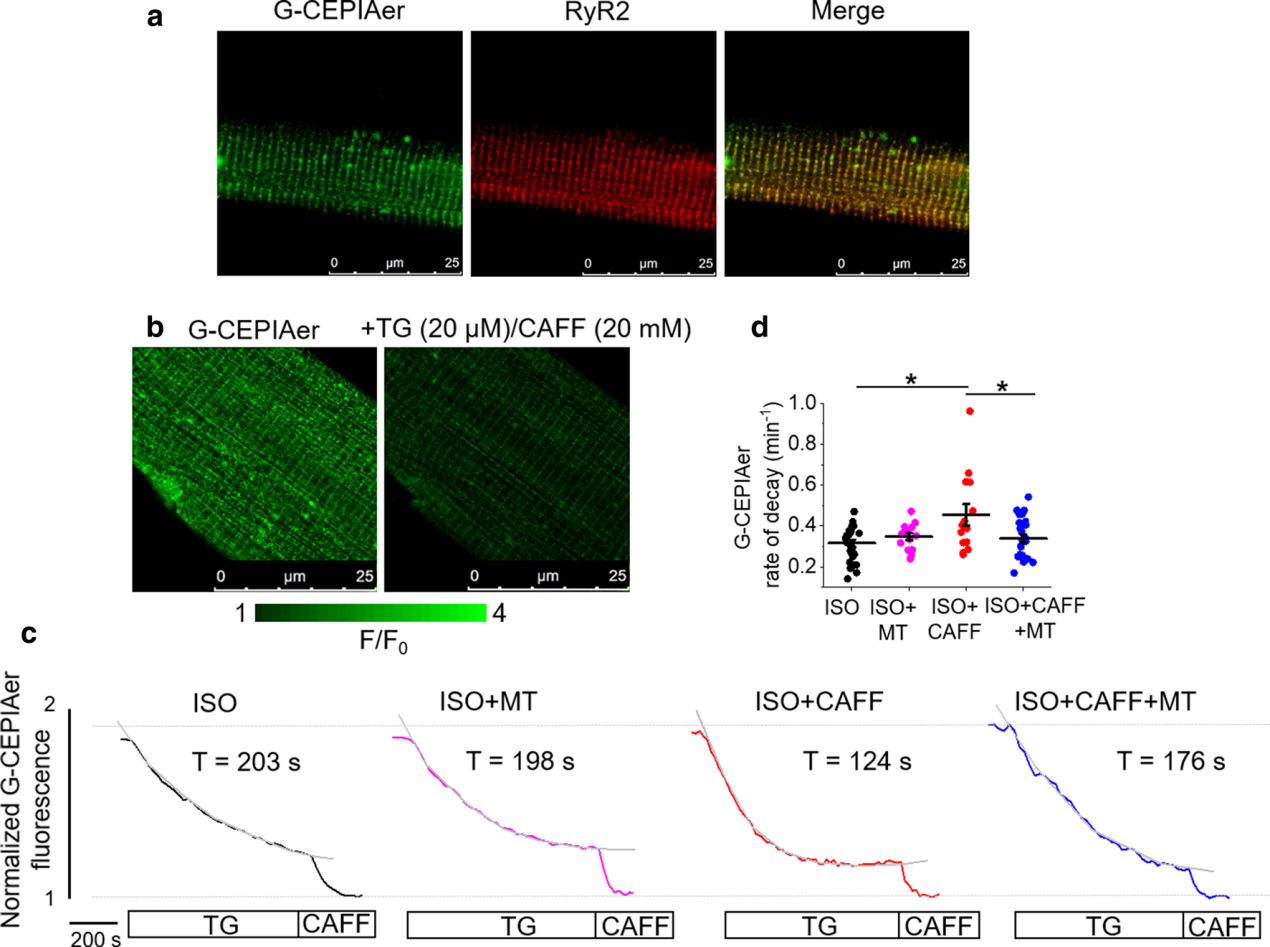
Mitochondrial ROS scavenging reduces caffeine-mediated SR Ca^2+^ leak in rat VMs. **a** Representative images of a cultured rat VM 48 h after adenoviral infection with G-CEPIAer intra-SR Ca^2+^ biosensor (left image, green). Using anti-RyR antibody, VMs were probed for RyR2 expression (center image, red). A merged image is shown on the right, indicating correct probe localization. **b** Representative images of infected VM treated with thapsigargin (20 µmol/L) and high-dose caffeine (20 mmol/L) to achieve minimal fluorescence, demonstrating G-CEPIAer sensitivity. **c** Representative time-dependent profiles (F/F_0_) of G-CEPIAer fluorescence from infected VMs. Myocytes were either treated with ISO (50 nmol/L), ISO and mitoTEMPO (MT, 20 μmol/L, 10 min pretreatment), ISO and caffeine (CAFF, 200 μmol/L), or ISO, CAFF and mitoTEMPO. Myocytes were exposed to SERCA inhibitor thapsigargin (TG, 10 μmol/L) after 1 min of recording, and the time constant of decay (τ, s) was calculated as a measure of SR Ca^2+^ leak. Signal was normalized to minimum fluorescence obtained by application of high-dose caffeine (10 mmol/L). The graph in d depicts mean data ± SEM for rate of decay (min^−1^). *N* = 5–9 animals, *n* = 14–26 VMs. *p < 0.05 vs. ISO group, **p < 0.05 vs. ISO + CAFF group, one-way ANOVA with Bonferroni post hoc *test*

To assess the effects of caffeine on [Ca^2+^]_SR_, we utilized genetic probe G-CEPIAer, a GFP-based biosensor spatially restricted to the SR, with a Kd ~ 672 µmol/L for Ca^2+^ [63]. After generating adenovirus encoding the sensor, isolated VMs were infected and cultured 36–48 h prior to experimentation. When expressed in VMs, G-CEPIAer has a striated SR patterning in the VM, and overlaps with the signal produced by RyR2 antibody, demonstrating correct cellular localization (Fig. 1a). To demonstrate probe sensitivity, VMs were treated with thapsigargin and high-dose caffeine to deplete the SR Ca^2+^ store and obtain minimum fluorescence (Fig. 1b).

To directly measure SR Ca^2+^ leak in VMs under β-adrenergic stimulation (ISO, 50 nmol/L), SR Ca^2+^ uptake via SERCa2a was inhibited by application of thapsigargin (20 µmol/L). G-CEPIAer fluorescence was normalized to minimal fluorescence signal obtained by application of high-dose caffeine (10 mmol/L) to empty the SR at the end of the experiment, as shown in the representative recordings (Fig. 1c). Application of low-dose caffeine to VMs induced a dramatic increase in SR leak rate (Fig. 1d).

To test whether the caffeine-induced acceleration of SR Ca^2+^ leak has a mito-ROS-dependent component, we tested whether the caffeine-mediated increase in RyR2 activity could be attenuated by scavenging of ROS with mitoTEMPO (20 µmol/L). Indeed, the increased rate of SR Ca^2+^ leak induced by caffeine was significantly attenuated by pretreatment of VMs with mitoTEMPO (Fig. 1c, d), indicative that increased mito-ROS emission may play a role in increased RyR2 activity. Pretreatment of VMs with mitoTEMPO did not have a significant effect on SR Ca^2+^ leak during stimulation with ISO alone.

### Increased SR Ca^2+^ leak is associated with augmented ROS emission and RyR2 oxidation

**Fig. 2:**
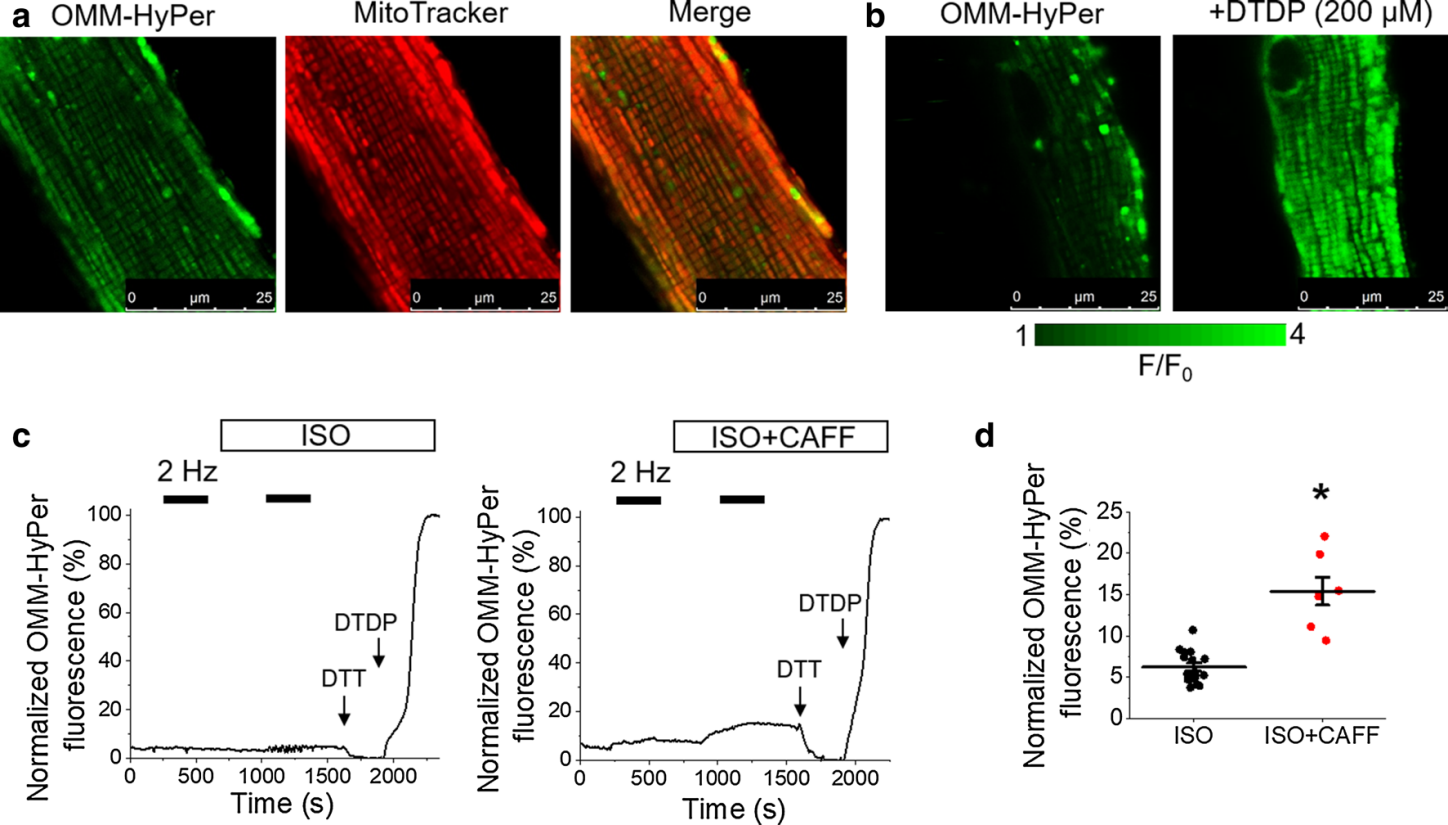
Increased SR Ca^2+^ leak increases mitochondrial ROS emission in rat VMs. **a** Representative image of a cultured rat VM 48 h after adenoviral infection with OMM-HyPer mito-ROS biosensor (left image, green). Myocytes were stained with mitochondrial dye MitoTracker Red (center image, red). A merged image is shown on the right, indicating correct probe localization. **b** Representative images of infected VM treated with DTDP (200 µmol/L) to achieve maximal fluorescence, demonstrating OMM-HyPer sensitivity. **c** Representative traces of OMM-HyPer fluorescence from infected VMs. Myocytes were paced at 2 Hz for 5 min (black bars), and treated with ISO (50 nmol/L, left trace) or ISO and caffeine (CAFF, 200 µmol/L, right trace) before further pacing. The signal was normalized to minimum fluorescence obtained by application of DTT (5 mmol/L) and maximum fluorescence by application of DTDP (200 µmol/L). The graph in **d** depicts mean data ± SEM for maximum normalized fluorescence (%) after pacing and application of ISO, or application of ISO and CAFF. *N* = 3–4 animals, *n* = 7–16 VMs. *p < 0.05 vs. ISO group, two-sample Student’s *t *test

Having demonstrated the protective effects of mito-ROS scavenging in VMs under conditions of enhanced SR Ca^2+^ leak, we next sought to visualize whether a caffeine-mediated increase in RyR2 activity leads to increased ROS emission. Using H_2_0_2_-sensitive fluorescent probe targeted to the outer mitochondrial membrane, OMM-HyPer [10, 32], ROS emission was assessed at the OMM of infected VMs (Fig. 2). Correct cellular localization of OMM-HyPer was confirmed by staining of mitochondria with far-red fluorescent indicator MitoTracker Red, as shown in Fig. 2a. Probe sensitivity is demonstrated in Fig. 2b, whereby VMs were treated with DTDP (200 µmol/L) to obtain maximum fluorescence.

As described in the methods, a standard pacing protocol was followed for organelle-targeted biosensors. After 5 min of recording, VMs were paced at 2 Hz for 5 min to apply the workload. After 2 min of pacing cessation, ISO (50 nmol/L) or ISO and low-dose caffeine (200 µmol/L) was added, and perfused for 5 min, followed by an additional pacing train for 5 min. Representative fluorescence recordings are shown in Fig. 2c. Signal was normalized to minimal fluorescence obtained by application of DTT (5 mmol/L) and maximal fluorescence by application of DTDP (200 µmol/L). The application of ISO and caffeine significantly increased OMM-HyPer signal vs. ISO alone (Fig. 2d), indicative that increased RyR2 activity can lead to increased ROS emission from the mitochondria.

**Fig. 3:**
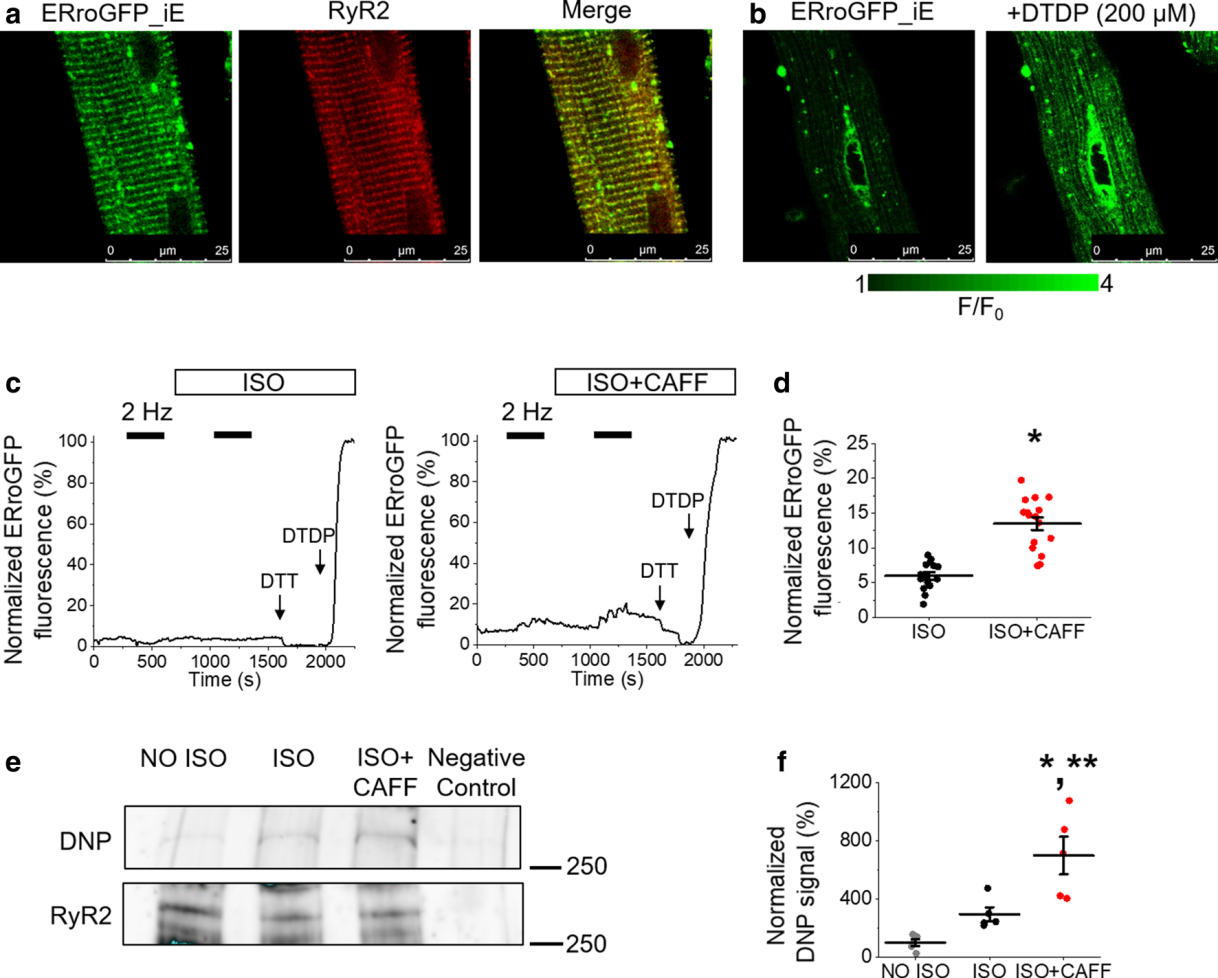
Enhanced RyR2 activity in rat VMs leads to SR oxidative stress and RyR2 oxidation. **a** Representative images of a cultured rat VM 48 h after adenoviral infection with ERroGFP_iE oxidative stress biosensor (left image, green). Using anti-RyR antibody, VMs were probed for expression of RyR2 (center image, red). A merged image is shown on the right, indicating correct probe localization. **b** Representative images of infected VM treated with DTDP (200 µmol/L) to achieve maximal fluorescence, demonstrating ERroGFP_iE sensitivity. **c** Representative traces of ERroGFP_iE fluorescence from infected VMs. Myocytes were paced at 2 Hz for 5 min (black bars) and treated with ISO (50 nmol/L, left trace) or ISO and caffeine (CAFF, 200 µmol/L, right trace). Signal was normalized to minimum fluorescence obtained by application of DTT (5 mmol/L) and maximum fluorescence by application of DTDP (200 µmol/L). The graph in **d** depicts mean data ± SEM for maximum normalized fluorescence (%) after pacing and application of ISO, or application of ISO and CAFF. *N* = 3–4 animals, *n* = 15–16 VMs. *p < 0.05 vs. ISO group, two-sample Student’s *t *test. **e** Immunoprecipitated RyR2 from freshly isolated rat VMs was immunoblotted for oxidation using DNP antibody. Representative images of DNP and RyR2 immunoprecipitation signal from VMs without treatment (NO ISO), treated with ISO, or ISO and CAFF. The graph in **f** depicts quantification of normalized DNP signal (%). *N* = 5 animals. *p < 0.05 vs. NO ISO group, **p < 0.05 vs. ISO group, one-way ANOVA with Bonferroni post hoc test

Mitochondria and the SR are closely situated in VMs, with bidirectional communication occurring between the two organelles [20, 25]. We therefore measured oxidative stress within the SR and in the vicinity of RyR2, using SR-targeted redox sensitive biosensor ERroGFP_iE [2, 32] (Fig. 3). The probe showed SR-like pattern of expression as evidenced by colocalization with RyR2 protein (Fig. 3a). Sensitivity of ERroGFP_iE is demonstrated in Fig. 3b, with application of DTDP to obtain maximal fluorescence. Similar results were obtained for those when using OMM-HyPer probe, whereby ISO plus caffeine treatment significantly increased ERroGFP_iE fluorescence vs. ISO alone (Fig. 3c, d), demonstrating that there is increased oxidative stress within the SR when RyR2 activity is enhanced.

To directly assess the oxidation status of RyR2 in VMs, the free thiol content of immunoprecipitated RyR2 was measured using the DNP antibody. Figure 3e, f demonstrates that treatment of VMs with ISO led to an increase in signal, but this was not significant. However, low-dose caffeine dramatically increased oxidation of RyR2 by sevenfold. This provides evidence that enhancement of RyR2 activity and SR Ca^2+^ leak can increase mito-ROS emission, increase deleterious oxidative stress within the SR and thus feedback on the redox status of the channel.

### Mito-Ca^2+^ and mito-ROS emission is modulated by RyR2 activity

**Fig. 4:**
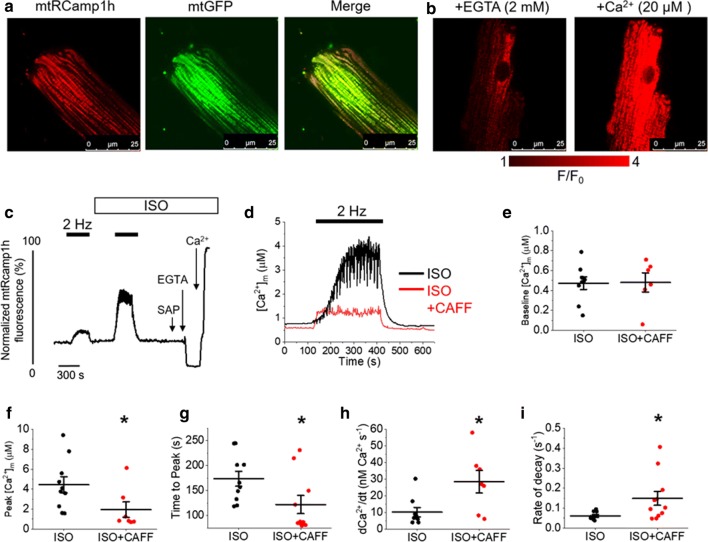
Enhancement of RyR2 activity in rat VMs diminishes the ability of mitochondria to accumulate and retain Ca^2+^. **a** Representative image of a cultured rat VM 48 h after adenoviral infection with mtRCamp1h mitochondrial Ca^2+^ biosensor (left image, red), and mitochondrial-targeted GFP (mtGFP, center image, green). A merged image is shown on the right, indicating correct probe localization. **b** Representative images of infected VM treated with EGTA (2 mmol/L) to achieve minimal fluorescence, and Ca^2+^ (20 µmol/L) to achieve maximal fluorescence, demonstrating mtRCamp1h sensitivity. **c** Representative trace of mtRCamp1h fluorescence from infected VM. Myocytes were paced at 2 Hz for 5 min (black bars), before treatment with ISO (50 nmol/L), or ISO and caffeine (CAFF, 200 µmol/L) and further pacing. Myocytes were saponin permeabilized (SAP) before treatment with EGTA (2 mmol/L) to obtain minimum fluorescence, followed by Ca^2+^ (20 µmol/L) to obtain maximal fluorescence. **d** Representative traces of [Ca^2+^]_m_ (µmol/L) during pacing in VMs treated with ISO (black line) or ISO and CAFF (red line). Graphs depict mean data ± SEM of ISO and ISO + CAFF groups for: **e** baseline [Ca^2+^]_m_ (μmol/L); **f** peak [Ca^2+^]_m_ (µmol/L); **g** time to peak [Ca^2+^]_m_ (s); **h** the first derivative of [Ca^2+^]_m_, indicative of maximal Ca^2+^ influx rate into the mitochondria during pacing (nM Ca^2+^ s^−1^); and **i** the rate of decay (s^−1^). *N* = 4 animals per group, *n* = 9 VMs per group. *p < 0.05 vs. ISO group, two-sample Student’s *t *test

We next investigated whether Ca^2+^ transfer from the SR to the mitochondria is involved in mito-ROS production. Using mitochondrial-matrix targeted biosensor mtRCamp1h, with a Kd ~ 1.3 µmol/L for Ca^2+^ [1, 32], we examined whether caffeine-mediated SR Ca^2+^ leak modulated global free mito-Ca^2+^ concentration ([Ca^2+^]_m_) in rat VMs (Fig. 4). After infection and culture of isolated VMs with mtRCamp1h adenovirus, expression of the probe within the mitochondrial matrix is observed. Correct intracellular targeting of mtRCamp1h was confirmed by co-infection with mitochondrial-targeted GFP adenovirus (Fig. 4a). Probe sensitivity is demonstrated in Fig. 4b. Application of calcium buffer EGTA was used to obtain minimal fluorescence, while Ca^2+^ (20 µmol/L) was used to obtain maximal fluorescence of saponin-permeablized VMs.

A representative recording is presented in Fig. 4c. A measurable increase in mtRCamp1h fluorescence occurs under conditions of enhanced workload, with field stimulation (pacing at 2 Hz, black bar). Fluorescence returns to baseline when field stimulation stops. Myocytes were paced using the standard 5 min, 2 Hz protocol before and after the application either ISO alone, or ISO and low-dose caffeine to induce SR Ca^2+^ leak. After this protocol, VMs were saponin permeabilized. Minimal (EGTA, 2 mmol/L) and maximal (Ca^2+^, 20 µmol/L) fluorescence signal was obtained, allowing for recalculation of signal to [Ca^2+^]_m_. As shown in Fig. 4d, [Ca^2+^]_m_ increases during pacing, with significantly reduced peak [Ca^2+^]_m_ when treated with ISO and low-dose caffeine, compared to ISO alone (Fig. 4f). The baseline [Ca^2+^]_m_ of VMs in the experimental groups is unchanged (Fig. 4e). This is suggestive that SR Ca^2+^ leak reduces total mito-Ca^2+^ retention. Application of ISO and caffeine significantly reduced the time to peak amplitude (Fig. 4g) and increased the first derivative of [Ca^2+^]_m_ (Fig. 4h). This indicates an increased maximal Ca^2+^ influx rate (nmol/L Ca^2+^ s^−1^) into the mitochondria during pacing. The rate of transient decay was significantly increased after caffeine treatment (Fig. 4i), demonstrating that caffeine also induces faster efflux of Ca^2+^ from the mitochondria that is evident when pacing stops.

**Fig. 5:**
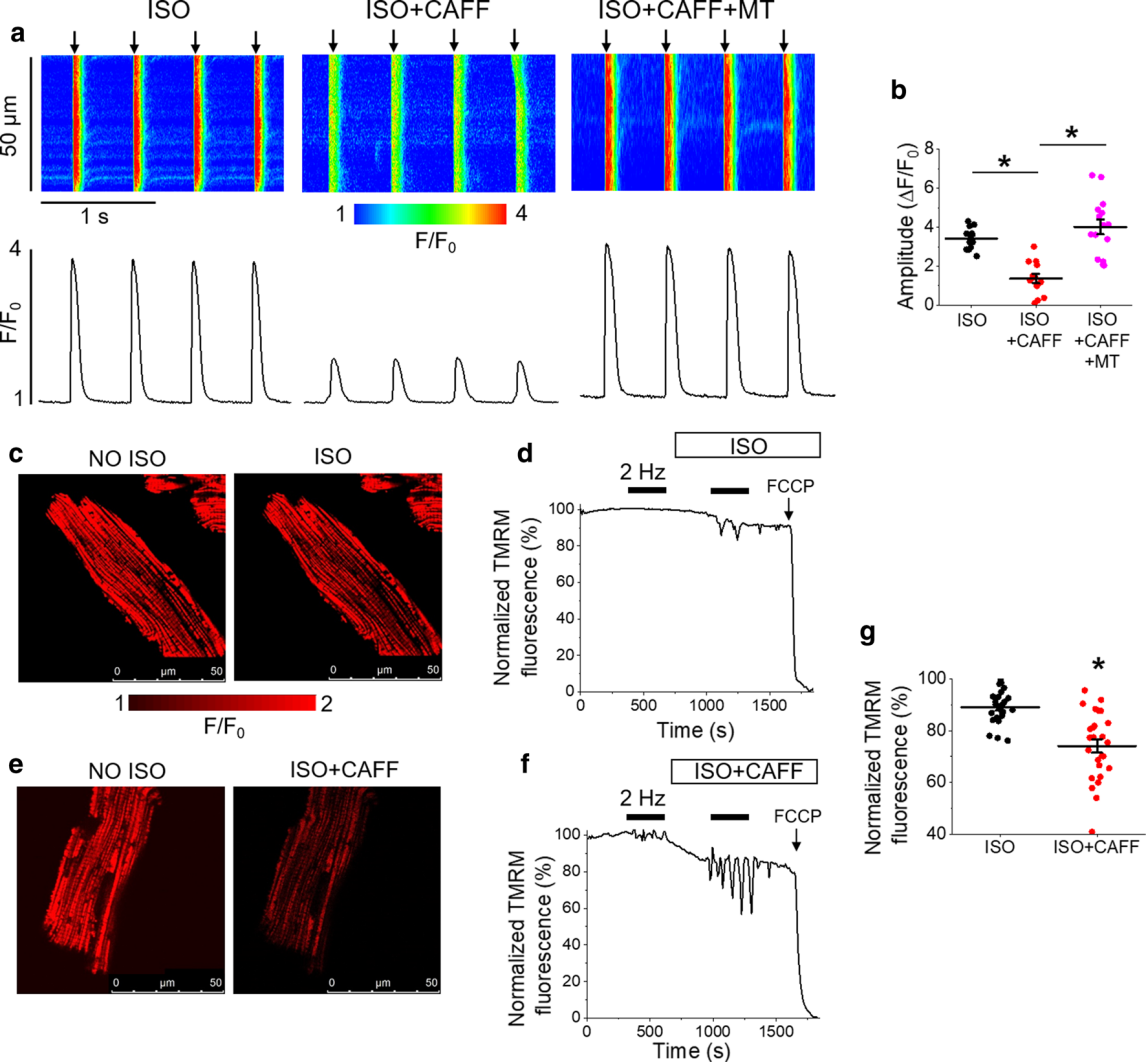
Enhancement of RyR2 activity reduces cytosolic Ca^2+^ transient amplitude and mitochondrial membrane potential. **a** Representative Fluo-3 fluorescence (F/F_0_) images (upper images) and profiles (lower images) of ISO treated (50 nmol/L) rat VM undergoing standard pacing protocol (arrows) of 2 Hz for 5 min, before and after treatment with low-dose caffeine (CAFF, 200 µmol/L) or mitoTEMPO (20 µmol/L) and caffeine. Line scan images were recorded during the last minute of pacing. The graph in b depicts mean data ± SEM for Ca^2+^ transient amplitude (Δ*F*/*F*_0_). *N* = 2–4 animals, *n* = 12–15 VMs. *p < 0.05, one-way ANOVA with Bonferroni post hoc test. **c** Representative images of a rat VM before and after treatment with ISO. Myocyte ΔΨm was monitored by TMRM fluorescence. **d** Representative recording of changes in ΔΨm in response to pacing at 2 Hz (black bar) for 5 min, followed by application of ISO and further pacing. The signal was normalized to minimum fluorescence obtained by application of FCCP (50 μmol/L) and represented as percentage of baseline. **e** Representative images of a TMRM-stained rat VM before and after treatment with ISO and CAFF. **f** Representative recording of changes in Δ*Ψ*m in response to pacing and application of ISO and CAFF. The graph in g depicts mean data ± SEM of minimum fluorescence after application of ISO or ISO and CAFF, and pacing. *N* = 3–4 animals, *n* = 26 VMs per group. *p < 0.05 vs. ISO group, two-sample Student’s *t *test

We next sought to explore how enhanced RyR2 activity reduces the capacity of mitochondria to accumulate and retain Ca^2+^. Myocytes loaded with fluorescent Ca^2+^ indicator Fluo-3 were subjected to the standard 5 min, 2 Hz pacing protocol (Fig. 5). Line scans were recorded during the last minute of pacing (Fig. 5a). Cytosolic Ca^2+^ transient amplitude was significantly reduced in VMs treated with ISO and low-dose caffeine, compared to ISO alone (Fig. 5b), which may explain the differences in [Ca^2+^]_m_ under these conditions. Pretreatment of VMs with mitoTEMPO attenuated the caffeine-mediated reduction in Ca^2+^ transient amplitude (Fig. 5a–b), indicative that enhanced mito-ROS is in part responsible for this effect.

Using cultured rat VMs stained with mitochondrial voltage-sensitive dye TMRM (20 μmol/L for < 1 min), we assessed whether increased RyR2 activity modifies mitochondrial membrane potential (ΔΨ_m_). Images in Fig. 5c show a cultured VM before and after application of ISO. A representative trace is shown in Fig. 5d, whereby signal was normalized to minimum fluorescence obtained by the application of carbonyl cyanide-p-trifluoromethoxyphenylhydrazone (FCCP, 50 μmol/L). Images in Fig. 5e show a cultured VM before and after application of ISO and low-dose caffeine, while a representative trace is shown in Fig. 5f. Treatment with ISO and caffeine significantly reduced TMRM fluorescence compared to treatment with ISO alone (Fig. 5g), suggestive that a reduction in ΔΨm may serve as a potential mechanism that contributes to [Ca^2+^]_m_ reduction under conditions of enhanced RyR2 activity.

**Fig. 6:**
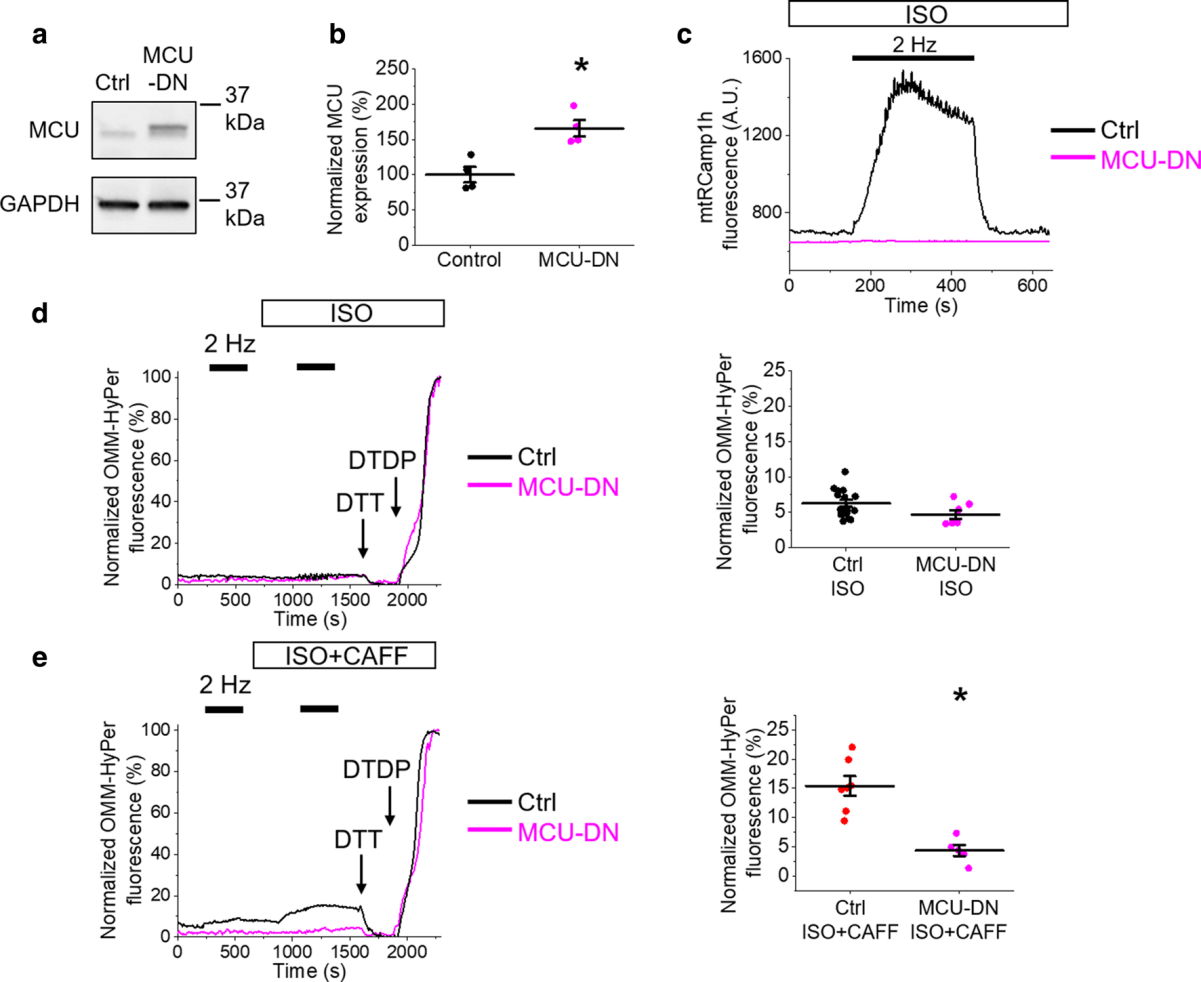
Inhibition of MCU Ca^2+^ uptake with MCU-DN reduces mito-ROS emission in rat VMs. **a** Representative western blots from VMs probed for MCU expression. The control lane (Ctrl) represents VMs infected with virus carrying an empty vector, and the MCU-DN lane represents VMs infected with virus carrying the dominant-negative MCU subunit. The graph in **b** depicts quantification of normalized MCU signal (%). *N* = 4 animals. *p < 0.05 vs. Ctrl group, two-sample Student’s *t *test. **c** Representative traces of mtRCamp1h fluorescence from control virus (black line) or MCU-DN virus (pink line) infected VMs. Myocytes were paced at 2 Hz for 5 min (black bar) after treatment with ISO (50 nmol/L) or ISO and low-dose caffeine (CAFF, 200 µmol/L). No mitochondrial Ca^2+^ uptake is observed with expression of MCU-DN. **d** Representative traces of OMM-HyPer fluorescence from infected VMs. Myocytes were paced at 2 Hz for 5 min (black bars), treated with ISO, and paced again at 2 Hz. The black line indicates control VM signal, while the pink line indicates MCU-DN infected VM. The signal was normalized to minimum fluorescence obtained by application of DTT (5 mmol/L) and maximum fluorescence by application of DTDP (200 µmol/L). The graph depicts mean data ± SEM for maximum normalized fluorescence (%) after pacing and application of ISO. *N* = 3–4 animals, *n* = 7–16 VMs. *p < 0.05 vs. Ctrl group, two-sample Student’s *t *test. **e** Representative traces of OMM-HyPer fluorescence from infected VMs. Myocytes were paced at 2 Hz for 5 min (black bars), treated with ISO and CAFF, and paced again at 2 Hz. The graph in e depicts mean data ± SEM for maximum normalized fluorescence (%) after pacing and application of ISO. *N* = 3 animals per group, *n* = 5–7 VMs. *p < 0.05 vs. Ctrl group, two-sample Student’s *t *test

To examine whether mito-ROS emission seen in the presence of low-dose caffeine is a process dependent on SR-mitochondria Ca^2+^ transfer, we utilized genetic inhibition of mito-Ca^2+^ uptake by adenoviral overexpression of the dominant-negative MCU pore subunit, MCU-DN [21]. Overexpression of MCU-DN in VMs is indicated via western blot, with increased protein signal indicated by anti-MCU antibody (Fig. 6a, b). To confirm inhibition of mito-Ca^2+^ uptake with MCU-DN overexpression, we co-expressed the mtRCamp1h biosensor. As demonstrated in Fig. 6c, MCU-DN prevented an increase in mtRCamp1h signal usually observed during pacing, indicating an inhibition of Ca^2+^ influx into mitochondria. Co-expression of OMM-HyPer biosensor revealed no changes in OMM-HyPer signal in VMs stimulated with ISO alone (Fig. 6d). As demonstrated in Fig. 6e, expression of MCU-DN significantly reduced OMM-HyPer fluorescence of ISO and caffeine-treated VMs when mito-Ca^2+^ uptake is inhibited (Fig. 6e). This supports the hypothesis in conditions of enhanced RyR2 activity, increased mito-ROS emission is a process dependent on influx of Ca^2+^ into the mitochondria.

### Augmented activity of RyR2 in CPVT VMs can be attenuated by scavenging of mito-ROS

**Fig. 7:**
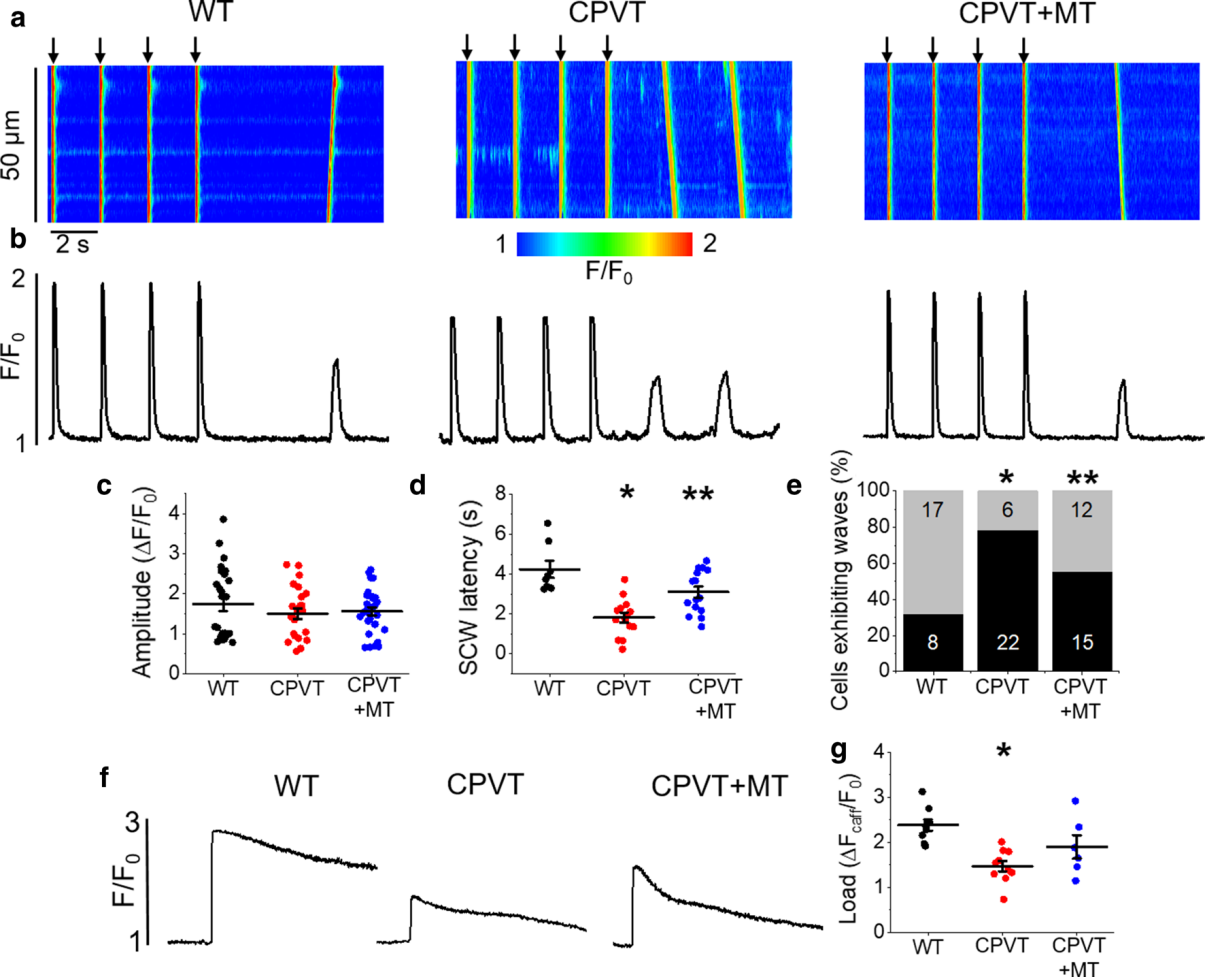
Proarrhythmic SCWs in CPVT mice VMs with genetically increased RyR2 activity can be attenuated by mito-ROS scavenging. **a** Representative confocal line scan images of Ca^2+^ transients and **b** Fluo-3 fluorescence (F/F_0_) profiles of ISO treated (100 nmol/L) WT and CPVT mouse VMs undergoing 0.5 Hz pace-pause protocol (arrows) to induce SCWs. Myocytes from CPVT mice were pretreated with mitoTEMPO (20 µmol/L, 20 min, CPVT + MT group). Graphs in **c**and **d** depict mean data ± SEM for WT, CPVT and CPVT + MT groups of Ca^2+^ transient amplitude (ΔF/F_0_) and SCW latency (seconds), respectively. *N* = 3 WT, 5 CPVT animals, *n* = 12–16 VMs. *p < 0.05 vs. WT group, **p < 0.05 vs. CPVT group, one-way ANOVA with Bonferroni post hoc test. **e** Graph depicting the percentage (%) of cells exhibiting waves. *N* = 3 WT, 5 CPVT animals, *n* = 20–25 VMs. *p < 0.05 vs. WT group, **p < 0.05 vs. CPVT group, Fisher’s exact test with Freeman-Halton extension. **f** Representative Fluo-3 fluorescence profiles (F/F_0_) of caffeine-induced Ca^2+^ transients (10 mmol/L) used to determine SR Ca^2+^ content. The graph in g depicts mean data ± SEM of caffeine-sensitive SR Ca^2+^ content (ΔF/F_0_). *N* = 3 WT, 3 CPVT animals, *n* = 4–8 VMs. *p < 0.05 vs. WT group, one-way ANOVA with Bonferroni post hoc test

We next sought to determine whether chronic genetically evoked increase in SR Ca^2+^ leak modulates mito-ROS and the oxidation status of RyR2, amplifying SR Ca^2+^ leak. We used a mouse CPVT model where chronic RyR2-mediated leak is evoked by ablation of the SR Ca^2+^ buffering protein calsequestrin [37]. Freshly isolated WT and CPVT VMs were loaded with the fluorescent Ca^2+^ indicator Fluo-3 AM and cytosolic Ca^2+^ transients were recorded in the presence of β-adrenergic receptor agonist ISO (100 nmol/L), subjecting VMs to a burst-pace pause protocol (0.5 Hz, 20 s). Representative images of Ca^2+^ transients and Fluo-3 AM fluorescence profiles (Δ*F*/*F*_0_) are shown in Fig. 7a and b, respectively. At low stimulation frequency, the Ca^2+^ transient amplitude did not show significant differences between CPVT and WT VMs (Fig. 7c). However, the latency of SCW was significantly reduced in diseased VMs (Fig. 7d). A reduced SCW latency is attributable to abnormal activity of RyR2 and serves as an indicator for the propensity of arrhythmogenic Ca^2+^ release [5, 16]. The percentage of CPVT VMs exhibiting waves was also significantly increased in comparison to controls (Fig. 7e), while the SR Ca^2+^ content was reduced (Fig. 7f, g). Besides reduced buffering capacity of the SR devoid of major Ca^2+^ buffer calsequestrin, this may indicate the hyperactivity of the RyR2 channel complex. To demonstrate that increased SR Ca^2+^ leak is mediated at least in part by mito-ROS, we pre-treated CPVT VMs with ROS scavenger mitoTEMPO (20 µmol/L, 20 min). This significantly increased SCW latency and restored SR Ca^2+^ content to near WT levels, suggesting that ROS scavenging can partially stabilize proarrhythmic RyR2 activity in CPVT VMs, similar to the effects of mitoTEMPO in rat VMS acutely challenged with caffeine (Figs. 1, 5a, b).

**Fig. 8:**
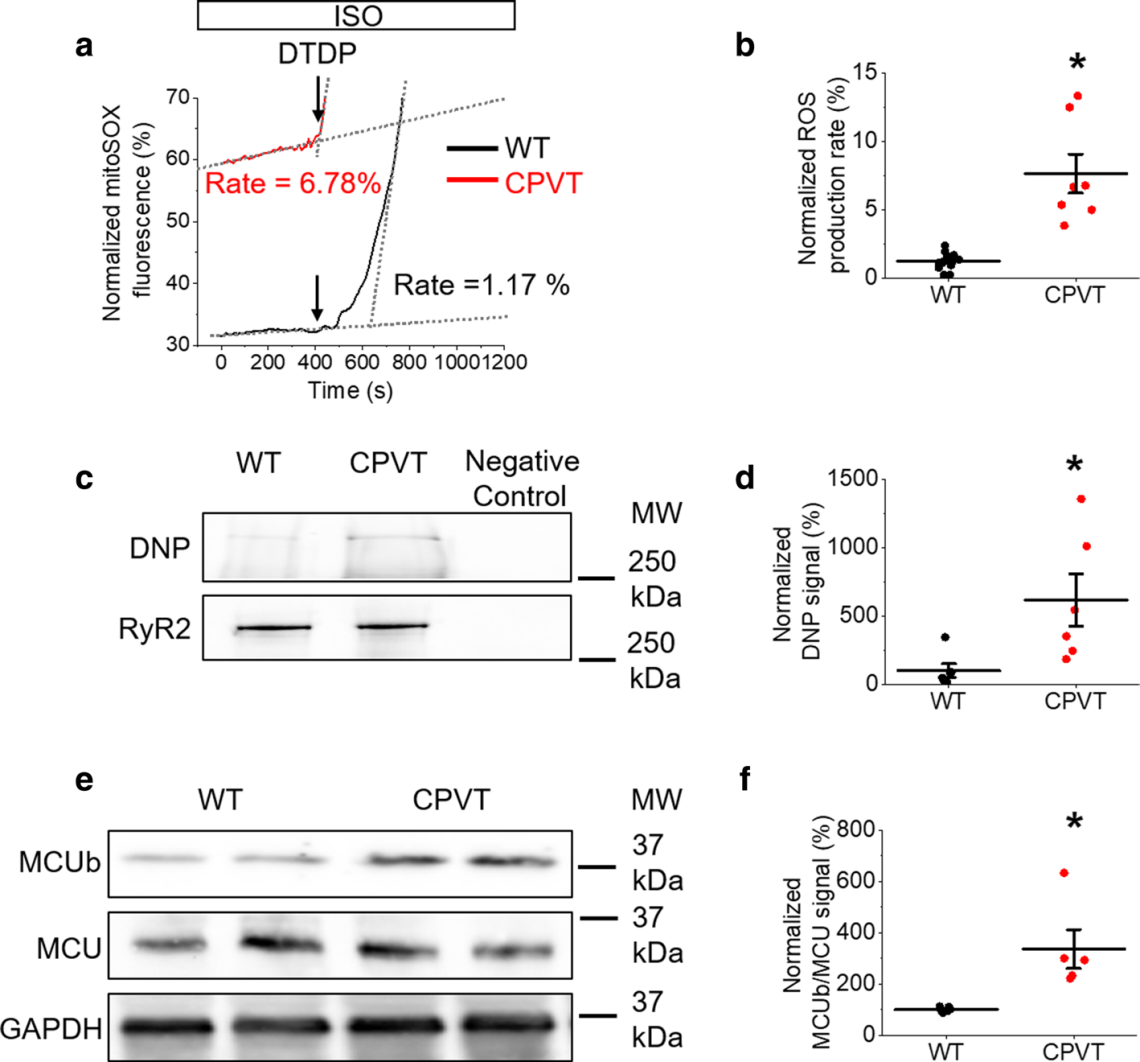
Myocytes of CPVT mice have increased mito-ROS emission rates and RyR2 oxidation in comparison to controls. **a** Representative recording of ROS production measured with MitoSOX in ISO-treated (100 nmol/L) WT and CPVT VMs. Signal was normalized to maximum fluorescence obtained on application of DTDP (200 µmol/L). The ROS production rate (%) was calculated by normalization to the maximum rate of ROS emission observed on application of DTDP, and rates for these representative recordings are indicated. The graph in **b** depicts mean data ± SEM for the normalized ROS production rate of WT and CPVT VMs. *N* = 3 WT, 5 CPVT animals, *n* = 7–13 VMs. *p < 0.05 vs. WT group, paired Student’s *t *test. **c** Representative images of immunoprecipitated RyR2 from freshly isolated ISO-treated WT and CPVT VMs, immunoblotted for RyR2 signal and oxidation using DNP antibody. The graph in **d** depicts quantification of normalized DNP signal (%). *N* = 6 animals per group. *p < 0.05 vs. WT group, two sample Student’s *t *test. **e** Representative western blots from WT and CPVT VMs probed for MCUb and MCU expression. The graph in **f** depicts quantification of normalized MCUb/MCU signal (%). *N* = 5 animals per group. *p < 0.05 vs. Ctrl group, two-sample Student’s *t *test

Using the mitochondria-specific ROS indicator MitoSOX, we show that ROS emission in CPVT VMs is significantly increased compared to controls (Fig. 8a, b). Furthermore, western blot revealed a significant increase in the oxidation status of RyR2 immunoprecipitated from diseased VMs (Fig. 8c, d), supporting the hypothesis that increased RyR2 activity in CPVT is partially modulated by leak-induced mito-ROS, as in rat VMs with a caffeine-induced increase in RyR2 activity (Figs. 2, 3).

Notably, CPVT does not cause massive functional changes at the whole heart and cellular levels [30], which would be expected if mitochondria function is substantially compromised. This points to the existence of compensatory mechanisms directed toward the reduction of SR-mitochondria Ca^2+^ transfer in conditions when SR Ca^2+^ leak is chronically increased. Indeed, as shown in Fig. 8e, f, CPVT is accompanied by a significant increase in expression levels of an inhibitory variant of MCU (MCUb; [41, 58]), and consequently a ~ 200% increase in the MCUb/MCU ratio. Taken together, our data suggest that hereditary enhancement in RyR2-complex activity affects mito-ROS production, exacerbating RyR2 dysfunction. Furthermore, although compensatory program to reduce SR-mitochondria Ca^2+^ transfer is activated in CPVT, this protection is clearly incomplete. Under β-adrenergic stimulation, upregulation of inhibitory MCUb does not prevent a surge in mito-ROS and the subsequent oxidation of RyR2.

## Discussion

Parallel changes in RyR2 activity and mito-ROS production are hallmark features in cardiac pathology such as HF, MI, aging, or diabetic-induced cardiomyopathy [4, 18, 22, 33, 34, 36, 64, 70]. We recently demonstrated that modulation of mito-Ca^2+^ efficiently regulates RyR2 activity via mito-ROS [32]. In the present study, we demonstrate that pharmacologically induced or genetic enhancement of RyR2 activity promotes mito-ROS production, and that SR-mitochondria Ca^2+^ transfer is essential for this process. We show that increased diastolic SR Ca^2+^ leak via hyperactive RyR2 channels drives further SR Ca^2+^ leak via mito-ROS, in an indirect, positive feedback process that is detrimental to intracellular Ca^2+^ handling in cardiac VMs.

### Leak begets leak

Robust control of RyR2-mediated SR Ca^2+^ release is vital for cardiac function. The amplitude of intracellular Ca^2+^ release increases to achieve greater contraction in conditions when the metabolic demands of the body are increased, i.e., stress [7]. This is largely attributed to the enhanced activity of SR Ca^2+^ ATPase and thereby higher intra-SR Ca^2+^ available during release. However, there is ample evidence suggesting that parallel changes in RyR2 activity play a significant role in this process. For example, it has been proposed that β-adrenergic stimulation increases “synchronization” of SR Ca^2+^ release from individual RyR2 clusters [62, 67] and the maximal rate of [Ca^2+^]_i_ rise [28]. Enhanced activity of RyR2 under β-adrenergic stimulation has been attributed to the direct effects of PKA phosphorylation [11, 53] or indirect effects via activation of CaMKII [19]. Importantly, prolonged exposure to β-adrenergic agonists was shown to evoke deleterious effects on Ca^2+^ cycling associated with enhanced RyR2 oxidation by mito-ROS and thereby its activity, resulting in th generation of pro-arrhythmic spontaneous Ca^2+^ waves [12, 13]. We previously showed that in chronic model of HF, enhanced phosphorylation levels of RyR2 are readily detectable relatively early during the course of pathological remodeling, while RyR2 oxidation becomes evident at much later stages [6]. Importantly, the combination of PKA/CaMKII-mediated phosphorylation and oxidation modifications of RyR2 led to more pronounced changes in RyR2-mediated SR Ca^2+^ leak and intracellular Ca^2+^ cycling than PKA/CaMKII-mediated phosphorylation alone [6, 40]. These studies strongly suggest that the effects of modulators of RyR2 activity can summate, and that primary modification of RyR2 can evoke secondary modification via systems of posttranslational control of RyR2 activity. To test this theory, we performed experiments in rat VMs with RyR2 activity directly enhanced by the pharmacological agent caffeine in the presence of β-adrenergic agonist ISO (Fig. 1). Parallel experiments with ROS biosensors targeted to mitochondria and the SR showed significant increases in ROS in the presence of caffeine (Figs. 2 and 3). This led to enhanced oxidation of RyR2 (Fig. 3). Application of mitochondria-specific ROS scavenger mitoTEMPO attenuated the effects of caffeine on RyR2-mediated SR Ca^2+^ leak (Fig. 1) and Ca^2+^ transient amplitude (Fig. 5), suggesting that initial insult produces a secondary effect on RyR2 function via a ROS-generating pathway involving mitochondria.

These results were further corroborated using calsequestrin2-null mouse model of RyR2-complex gain of function [37]. Western blot analysis using samples prepared from mouse hearts exposed to ISO shows significant increase in RyR2 oxidation in CPVT (Fig. 8c, d), while measurements of mito-ROS with mitoSOX in acutely isolated VMs demonstrate an eightfold increase in the rate of ROS production by mitochondria (Fig. 8a, b). Existing evidence of secondary posttranslational modulation of RyR2 activity in CPVT VMs includes increased phosphorylation of the channel by PKA [49] and CaMKII [45], while RyR2 oxidation was not previously shown. However, our findings are in line with a recent report demonstrating enhanced RyR2 oxidation and mito-ROS emission in a mouse model with chronic SR Ca^2+^ leak induced by phosphomimetic RyR2 mutation [60]_._ Incubation of ISO-challenged CPVT VMs with mitoTEMPO restored SR Ca^2+^ content and reduced spontaneous Ca^2+^ release (Fig. 7), which implies that oxidation of RyR2 secondary to genetically evoked gain of function of SR Ca^2+^ release plays a key role in Ca^2+^-dependent arrhythmia.

### Mitochondrial [Ca^2+^] and ROS

The close proximity of mitochondria and the SR allows for bidirectional communication between the organelles, whereby production of ATP by mitochondria can be closely coupled to RyR2-mediated Ca^2+^ release and the energy demand of the myocyte [20, 25]. Activity of several mitochondrial enzymes involved in oxidative phosphorylation depends on [Ca^2+^], thereby enhanced mitochondrial Ca^2+^ influx is thought to promote electron transport and ATP production [52]. However, increased activity of electron transport chain results in enhanced mito-ROS production, which can overcome intracellular antioxidant defenses and produce cell-wide deleterious effects [9, 29]. This is most evident during ischemia–reperfusion when mito-Ca^2+^ overload was directly linked to a massive surge in ROS [39, 51]. However, in other pathological conditions accompanied by enhanced catecholaminergic drive such as HF, the link between mito-[Ca^2+^] and rate of mito-ROS production remains a subject of controversy. Indeed, in guinea pig model of HF and rat model of hypertrophy, the levels of mitochondria ROS were shown to be increased, while levels of [Ca^2+^]_m_ dramatically reduced [38, 46, 48]. These data strongly suggest that the [Ca^2+^]/ROS relationship in mitochondria is not linear. It appears that in the absence of a catastrophic event such as ischemia–reperfusion, excessive Ca^2+^ accumulation can be effectively limited by robust control mechanisms, which clearly fail to stop the generation of mito-ROS. In the present work, we for the first time directly tested how acute enhancement of RyR2 activity affects intra-mitochondrial [Ca^2+^]. Experiments using intra-mitochondrial Ca^2+^ biosensor mtRCamp1h showed that periodic pacing in the presence of ISO induces mito-Ca^2+^ accumulation (Fig. 4). Pharmacological facilitation of RyR2 activity with low-dose caffeine to mimic HF phenotype produces a decrease in peak [Ca^2+^]_m_, an increase in the maximum rate of mito-Ca^2+^ loading during pacing, and an accelerated loss of [Ca^2+^] upon pacing cessation. Phenomenologically, these results are consistent with a previously reported loss of mito-Ca^2+^ in disease models with enhanced RyR2 activity, i.e., guinea pig HF and rat model of cardiac hypertrophy induced by pressure overload [32, 48]. Notably, the net reduction in [Ca^2+^]_m_ could be explained by a decrease in systolic [Ca^2+^]. Moreover, the caffeine-induced RyR2 leak appears to facilitate mito-Ca^2+^ loss, as shown in Fig. 4. Our experiments using voltage-sensitive indicator showed ΔΨ m depolarization in the presence of low-dose caffeine (Fig. 5c, d), which is expected to reduce the driving force for mito-Ca^2+^ uptake and can indicate mPTP opening, promoting rapid Ca^2+^ extrusion [15, 26, 35, 50, 69]. This can at least partially explain the reduction in net [Ca^2+^]_m_ in conditions with enhanced RyR2-mediated SR Ca^2+^ leak. Increased Ca^2+^ extrusion by the mitochondrial Na^+^/Ca^2+^/Li^+^ exchanger (NCLX) may also play a role in reducing net [Ca^2+^]_m_ during enhanced SR Ca^2+^ leak, as seen in conditions of cytosolic Na^+^ overload [38, 48].

Importantly, our results contradict the findings obtained in several models with hyperactive RyR2 channels. The Ca^2+^ content of isolated mitochondria was shown to be increased in mice with phosphomimetic RyR2 mutation [60] and in aging human hearts [59]. An explanation for this discrepancy could be that our measurements of free [Ca^2+^]_m_ with a genetically targeted biosensor in live VMs do not reflect total mito-Ca^2+^ accumulation, because we are not tracking Ca^2+^ complexed with phosphates. However, according to Chalmers and Nicholls [17, 54], free mito-Ca^2+^ must first be increased to promote the formation of Ca^2+^-phosphate complexes. A more likely scenario is that differences in cellular [Na^+^] between control and diseased VMs, and thereby changes in NCLX-mediated mito-Ca^2+^ efflux, are omitted upon mitochondria isolation. Furthermore, the reduction of cytosolic Ca^2+^ transient amplitude and [Ca^2+^] available for uptake observed in live VMs are not directly mimicked in in vitro studies. At the same time, our data demonstrating an increase in maximal mito-Ca^2+^ uptake rate (Fig. 4h) supports the hypothesis that MCU activity is increased, likely due to oxidation of the complex [23]. This may contribute to an increased [Ca^2+^] in isolated mitochondria from samples with leaky RyR2s. Future studies using protocols that match mito-Ca^2+^ fluxes in live cells and isolated preparations are needed to reconcile this controversy.

### The role of SR-mitochondria Ca^2+^ transfer in mito-ROS production

It is well established that increase of oxygen consumption by mitochondria under β-adrenergic stimulation is a Ca^2+^-dependent process [55]. Increased Ca^2+^ release in the presence of β-adrenergic agonists stimulates contraction and accelerates expenditure of ATP, decreasing the ATP/ADP ratio and thus promoting generation of ATP by mitochondria. The by-product of this process will be mito-ROS, which can lead to RyR2 oxidation and consequently generation of spontaneous Ca^2+^ waves. Indeed, Bovo et al. [13] recently showed that the presence of blebbistatin to specifically inhibit contraction substantially reduced oxidative stress in VMs under prolonged exposure to β-adrenergic agonist. However, this data does not fully exclude the potential role of direct SR-mitochondria Ca^2+^ transfer in modulation of mitochondria function and production of mito-ROS. Our recent study revealed that facilitation of mito-Ca^2+^ influx using pharmacological activators of MCU in the presence of ISO fails to increase mitochondria Ca^2+^ loading, and, similar to the effects of low-dose caffeine, depolarizes ΔΨm [32]. Likewise, both caffeine to increase RyR2 activity (Figs. 2 and 3) and MCU agonists accelerate production of mito-ROS. Previously, we also showed that inhibition of MCU was sufficient to reduce mito-ROS in VMs from hypertrophic rats [32]. Using the dominant-negative MCU variant, we obtained additional evidence that in the presence of caffeine, SR-mitochondria Ca^2+^ transfer is necessary for the mito-ROS surge (Fig. 5). Further indirect evidence that SR-mitochondria Ca^2+^ transfer plays a key role in disease-related mitochondria dysfunction was obtained by measuring the expression levels of MCU and its inhibitory variant MCUb in the hearts of CPVT mice (Fig. 8e, f). Importantly, recent electron microscopy studies in a mouse model of RyR2 loss of function demonstrated a substantial increase in tunneling between mitochondria, highlighting the responsiveness of the organelle to disturbances in SR Ca^2+^ release in an attempt to preserve mitochondrial function and energy supply [43]. A significant increase in MCUb/MCU ratio in CPVT suggests that RyR2 hyperactivity facilitates adaptive remodeling directed toward the reduction of mito-Ca^2+^ uptake.

## Limitations

Sarcoplasmic reticulum Ca^2+^ leak-induced changes in intracellular ROS, [Ca^2+^]_m_ and ΔΨ_m_ may produce secondary effects on other key components of intracellular Ca^2+^ handling machinery including SERCa2a, Na^+^/Ca^2+^ exchanger and L-type Ca^2+^ channels [8, 33, 68]. In the present study, we did not measure changes in activities of these complexes in caffeine-treated rat VMs or CPVT mouse VMs, nor did we measure intracellular [ATP]. Notably, our previous data demonstrates lack of changes and/or effects of ROS scavenging on activities of these complexes in aged rabbit VMs and canine HF and MI VMs with leaky RyR2 channels [4–6, 18]. This supports the hypothesis that mito-ROS-mediated, self-imposed enhancement of SR Ca^2+^ leak is the major contributor to defective Ca^2+^ homeostasis in conditions with hyperactive RyR2s.

## Conclusion

Taken together, our results suggest that both acute and inherited increase in activity of RyR2 evoke multiple mechanisms that protect mitochondria from Ca^2+^ overload. However, under conditions mimicking stress, these mechanisms fail to fully alleviate mito-Ca^2+^-dependent increase in mito-ROS, resulting in oxidation of RyR2. This further increases channel activity and exacerbates defective intracellular Ca^2+^ homeostasis.

## Abbreviations

ΔΨ_m_: Mitochondrial membrane potential
[Ca^2+^]_i_: Intracellular calcium concentration
[Ca^2+^]_m_: Mitochondrial calcium concentration
[Ca^2+^]_SR_: Sarcoplasmic reticulum calcium concentration
CAFF: Caffeine
CPVT: Catecholaminergic polymorphic ventricular tachycardia
HF: Heart failure
ISO: Isoproterenol
MCU: Mitochondrial Ca^2^^+^ uniporter
MCU-DN: Dominant negative subunit of mitochondrial Ca^2^^+^ uniporter
Mito-Ca^2+^: Mitochondrial Ca^2+^
Mito-ROS: Mitochondria-derived reactive oxygen species
NCLX: Na^+^/Ca^2+^+/Li^+^ exchanger
OMM: Outer mitochondrial membrane
ROS: Reactive oxygen species
RyR2: Ryanodine receptor
SCW: Spontaneous Ca^2+^ wave
SERCa2a: Sarco/endoplasmic reticulum Ca^2+^-ATPase
SR: Sarcoplasmic reticulum
VM: Ventricular myocyte
VT/VF: Ventricular tachycardia/ventricular fibrillation
WT: Wild type
